# Beta frequency binaural beats combined with preferred music enhance combat performance and recovery responses in amateur kickboxers: a randomized crossover trial

**DOI:** 10.3389/fpsyg.2025.1636856

**Published:** 2025-09-16

**Authors:** Nidhal Jebabli, Manar Boujabli, Mariem Khlifi, Nejmeddine Ouerghi, Anissa Bouassida, Abderraouf Ben Abderrahman, Wissem Dhahbi, Roland van den Tillaar

**Affiliations:** ^1^Research Unit: Sport Sciences, Health and Movement, High Institute of Sport and Physical Education of Kef, University of Jendouba, Kef, Tunisia; ^2^Perf-Up Rehabilitation Center, Doha, Qatar; ^3^Faculty of Medicine of Tunis, Rabta Hospital, LR99ES11, University of Tunis El Manar, Tunis, Tunisia; ^4^High Institute of Sport and Physical Education of Gafsa, University of Gafsa, Gafsa, Tunisia; ^5^Higher Institute of Sport and Physical Education of Ksar-Said, University of Manouba, Manouba, Tunisia; ^6^Training Department, Qatar Police Academy, Police College, Doha, Qatar; ^7^Department of Sports Science and Physical Education, Nord University, Levanger, Norway

**Keywords:** fast tempo music, binaural tone frequencies, activity profile, psychophysiological responses, combat sports

## Abstract

**Background:**

While binaural beats and preferred music demonstrate established ergogenic effects independently, their synergistic potential during combat sports recovery remains unexplored. This study aimed to investigate the combined effect of 15 Hz beta-frequency binaural beats with preferred music during inter-round recovery enhances striking performance and psychophysiological responses in kickboxing athletes.

**Methods:**

Nineteen amateur kickboxers (age: 21.5 ± 1.8 years; body mass: 68.7 ± 5.3 kg; height: 1.80 ± 0.08 m) completed simulated combat under three randomized crossover conditions using a Latin square randomization design: preferred music (PM), 15 Hz binaural beats with preferred music (15 Hz-BPM), and control. Measurements included striking indices (frequency, velocity), cardiovascular responses, rating of perceived exertion (RPE), feeling scale, and post-exercise blood lactate. The kickboxers could not be blinded due to the inherent auditory nature of the beta-frequency binaural beats.

**Results:**

Results demonstrated significant main and interaction effects for striking performance and physiological markers (*p* < 0.001, *η*^2^_p_ ≥ 0.29), indicating large effect sizes across primary outcomes. In addition, significant effects of round condition (*p* ≤ 0.027) for psychological parameters, while only a significant interaction effect was found for feeling scale and upper body RPE (*p* ≤ 0.005). Post-hoc analyses revealed that 15 Hz-BPM produced substantial improvements in striking frequency (*η*^2^_p_ = 0.29–0.33) and peak velocity compared to music-only and control conditions, with effect sizes indicating practically meaningful performance enhancements. Similarly, our results showed a significant improvement for heart rate, feeling scale and a significant decrease for RPE and lactate values post-round in the 15 Hz-BPM than in the other conditions.

**Conclusion:**

Combined preferred music and 15 Hz binaural beats during inter-round recovery enhanced striking performance and psychophysiological responses compared to single interventions or control conditions. These results suggest potential use of binaural beats and preferred music for performance optimization in amateur combat sports training.

## 1 Introduction

Kickboxing performance relies on the integration of tactical-technical proficiency with optimal physical and psychological preparation, where success depends on executing precise strikes while managing fatigue across multiple rounds (Buse and Santana, 2008). For amateur kickboxers, bouts are typically 3 to 12 rounds of 2 to 4 min with 1 to 2 min of recovery in between (Slimani et al., 2017). Professional kickboxers develop physical qualities such as endurance, strength, explosiveness, coordination, and agility (Slimani et al., 2017). Kickboxing requires more anaerobic than aerobic energy reserves, with increased recovery time between rounds needed to avoid fatigue and maximize performance (Rydzik and Ambroży, 2021).

Inter-round recovery, taking about 1 min, is the time for energy store replenishment, mainly of ATP-CP resynthesis and reoxygenation of the muscle (Campos et al., 2012). Lactate can be reduced, and physiological parameters stabilized through recovery strategies such as hydration, cooling, and rapid massage. However, these strategies remain insufficient and do not fully allow for the complete restoration of physiological capacities (Bogdanis et al., 1996; Guidetti et al., 2002).

As a result, psychological techniques are increasingly being emphasized as additional recovery strategies, especially during short breaks between rounds in combat sports. Listening to music, in particular, has been recognized for its potential in ergogenic, psychological and psychophysiological functions (Ballmann, 2021).

Theoretically, music has long been known for its ergogenic effects at various points before, during, and after exercise. These ergogenic mechanisms operate through distinct pathways: attenuation of peripheral fatigue signals (Diehl et al., 2023), enhancement of motor unit recruitment efficiency (Centala et al., 2020), modulation of prefrontal cortical activity, optimization of psychological states including mood (Jebabli et al., 2025), attentional focus (Patania et al., 2020), and self-efficacy (Pettit and Karageorghis, 2020). These findings suggest that music is a very effective tool for optimizing physical, cognitive, and affective restoration to both performance and recovery. However, few studies have directly addressed its role during exercise recovery in combat sports (Jones et al., 2017; Karageorghis et al., 2021).

Despite these findings, their interaction with musical characteristics, including tempo (slow, fast), frequency (in Hz), volume (in dB), listening mode (monaural, binaural), and listening time (before, during exercise), remains unknown. Their functions in sports science have been studied separately. For example, Jebabli et al. (2025) observed that listening to preferred music with frequencies around 440 Hz and 432 Hz, during warm-up, improve physical performance intermittent kickboxing anaerobic speed test, positive mood with a potential dissociation from discomfort during the test for both sexes.

During recovery periods, Jones et al. (2017) demonstrated that listening to fast-tempo music (of 125–135 bpm) during 3-min recovery period between high intensity interval training sets improved emotional state without significantly impacting cardiorespiratory recovery. Similarly, Karageorghis et al. (2021) discovered that listening to moderate-tempo recovery music (120–125 bpm) for 90 s as part of a high intensity interval training protocol decreased perceived exertion and supported training compliance, without differential heart rate measurement.

These studies support the importance of hearing stimuli during recovery but also point out that more studies are needed to address how such stimuli can more effectively enhance physiological recovery.

In this regard, binaural beats, with different frequency range, are starting to be recognized as innovative approaches to neuromodulation of cognitive and emotional states (Ingendoh et al., 2023).

In fact, binaural beat music involves presenting two slightly different auditory frequencies to each ear (e.g., 440 Hz and 425 Hz) to create a difference in perceived frequency (e.g., 15 Hz) that stimulates brain activity (Sudre et al., 2024). Also, binaural brainwaves are rhythmic patterns of neuronal activity, induced by synchronized electrochemical firing of populations of neurons in the central nervous system (Padulo et al., 2013; Chaieb et al., 2015).

Relative to alpha sub-bands (e.g., 8–12 Hz) or theta ranges (4–8 Hz) that tend to be associated with emotion or recall, the use of 15 Hz binaural beats in beta frequency is a promising method to improve neurocognitive functioning in cases of mental fatigue. Literature has shown that listening to 15 Hz binaural stimulation can improve cognitive functioning, working memory, attention and the efficiency of cortical networks during demanding cognitive tasks by facilitating brain connectivity (Beauchene et al., 2016; Colzato et al., 2017; Wang et al., 2022; Sudre et al., 2024). Neurophysiologically, 15 Hz beta binaural beats correspond to sensorimotor rhythm frequencies that optimize cortical arousal and enhance attentional focus through neural entrainment in the prefrontal and sensorimotor regions (Beauchene et al., 2016; Colzato et al., 2017; Sudre et al., 2024).

To our knowledge, only Wang et al. (2024) have reported that listening to fast-tempo music with 30 Hz beta binaural beats as a warm-up had no impact on repeated sprint ability test performance in young male and female soccer players.

To resume, it is necessary to note that the effect of binaural beats have been a relatively less studied area in sport (Wang et al., 2024). However, its integration with preferred music has not been evaluated in combat sports recovery contexts.

Therefore, the purpose of this study was to examine how the activity profile and psychophysiological reactions in a simulated kickboxing bout were affected when listening to favorite music embedded with a 15 Hz binaural beat both before and during inter-round recovery. We hypothesized that 15 Hz binaural beats combined with preferred music would produce: (i) 10–15% increases in striking frequency based on attention enhancement effects (Beauchene et al., 2016; Colzato et al., 2017); (ii) metabolic efficiency improvements reflected in lactate dynamics (Ballmann, 2021); (iii) enhanced affective responses through mood modulation (Engelbregt et al., 2021) and reduced perceived exertion via attentional dissociation mechanisms (Ouergui et al., 2023a; Ouergui et al., 2023b; Boujabli et al., 2024), compared to single-intervention and control conditions.

## 2 Methods

### 2.1 Study design

Prior to the experimental procedures, the kickboxers were thoroughly familiarized with all test procedures, instruments, and equipment in order to minimize learning effects.

This study is a randomized repeated measures crossover design examining the effect of listening to preferred music or beta binaural beats preferred music during pre and inter-round recovery on technical, physical and psychophysiological responses in kickboxing combat simulation on the punching bag. Kickboxers were exposed to three conditions: (1) listening to preferred music (PM) during pre- and inter-round recovery, (2) listening to 15 Hz (beta) binaural beats preferred music (15 Hz-BPM) during pre- and inter-round recovery and (3) neutral self-talk with no-music (control). During familiarization (2 weeks pre-testing), kickboxers became acquainted with all procedures while anthropometric data (height, weight, body mass index - BMI) were collected.

**Figure 1:**
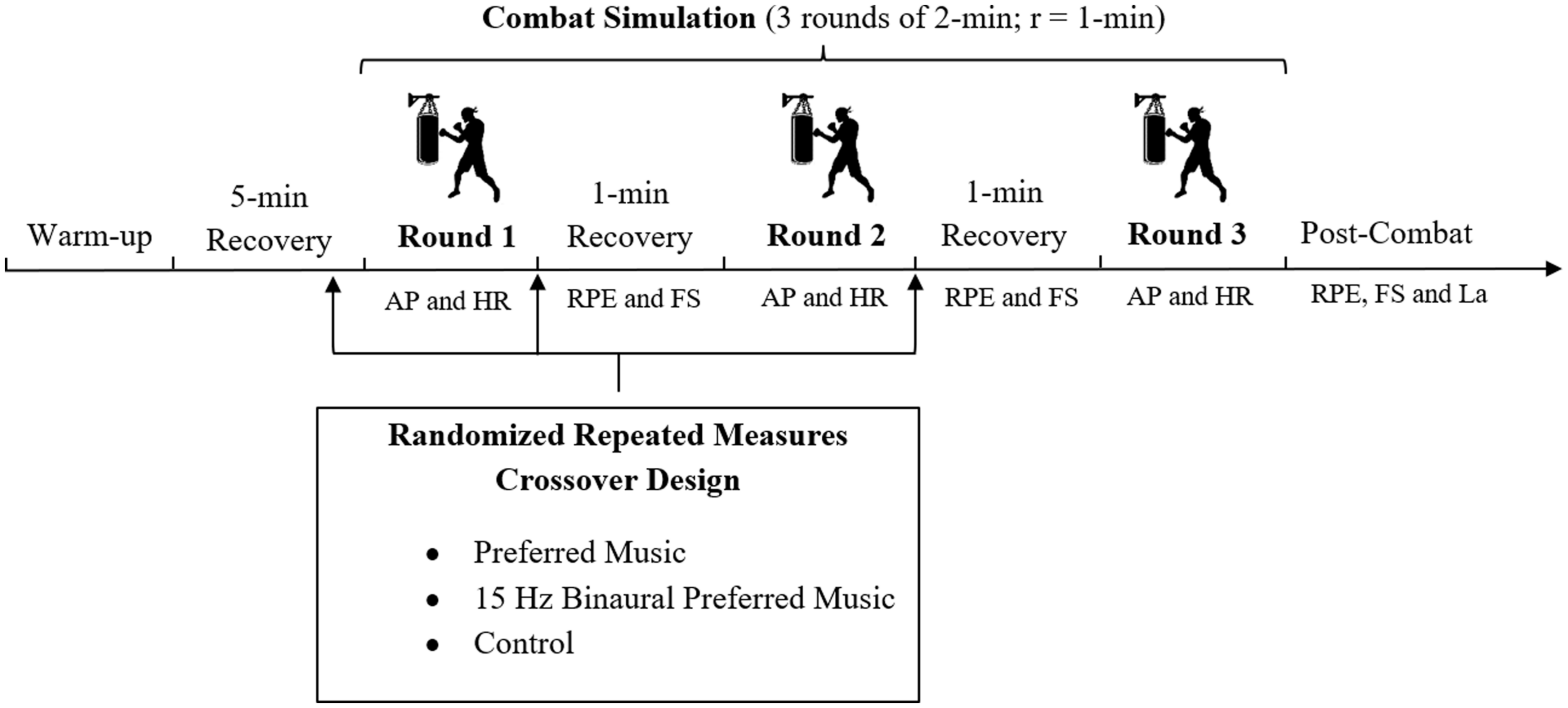
Experimental design. AP, activity profile; HR, heart rate; RPE, rating of perceived exertion; FS, feeling scale; La, lactate; r = 1-min passive recovery.

During the experimental period, the simulated kickboxing bout in each condition was performed separately for each kickboxer, in separate sessions, separated by 48 h of recovery. Condition order was randomized using a Latin square design to control for order effects, with each kickboxer assigned to one of six possible condition sequences (PM-15 Hz-BPM-Control, PM-Control-15 Hz-BPM, etc.) through computer-generated random allocation. Before each test session, the kickboxers performed a standardized 10-min warm-up program, according to van den Tillaar et al. (2019), consisting of 5 min of jogging (60–65% of maximum heart rate), 4 min of running (three 60-m runs at 75, 85, and 95% of self-estimated maximum intensity; recovery: 1 min between each of the three runs), and 1 minute of lateral movements and dynamic stretching. These sessions were followed by 2 minutes of passive recovery. All sessions occurred at consistent times (17.00 ± 1 h) in the same facility (ambient temperature: 23–25 °C) to control diurnal performance variations. Kickboxers were asked to refrain from any strenuous exercise during the experimental protocol period. They were also asked to maintain their hydration, dietary, and sleep habits, and to refrain from any consumption of ergogenic products (e.g., caffeine, vitamins) in the 24 h preceding each session. Figure 1 describes the experimental design.

### 2.2 Participants

*A priori* power analysis was conducted using G*Power software (Version 3.1.9.4, University of Kiel, Kiel, Germany) with the F test family (ANOVA: repeated measures, within factors). The sample size calculation was based on a statistical power of 0.80, a significance level of 0.05, and an effect size of *f* = 0.31, derived from previous music-exercise interventions in combat sports showing moderate-to-large effects on performance variables (Ouergui et al., 2023a; Jebabli et al., 2025). This effect size represents the anticipated difference between conditions based on meta-analytic evidence for music interventions in high-intensity exercise (Slimani et al., 2017). The analysis indicated that 19 kickboxers were needed to achieve 80% power.

**Figure 2:**
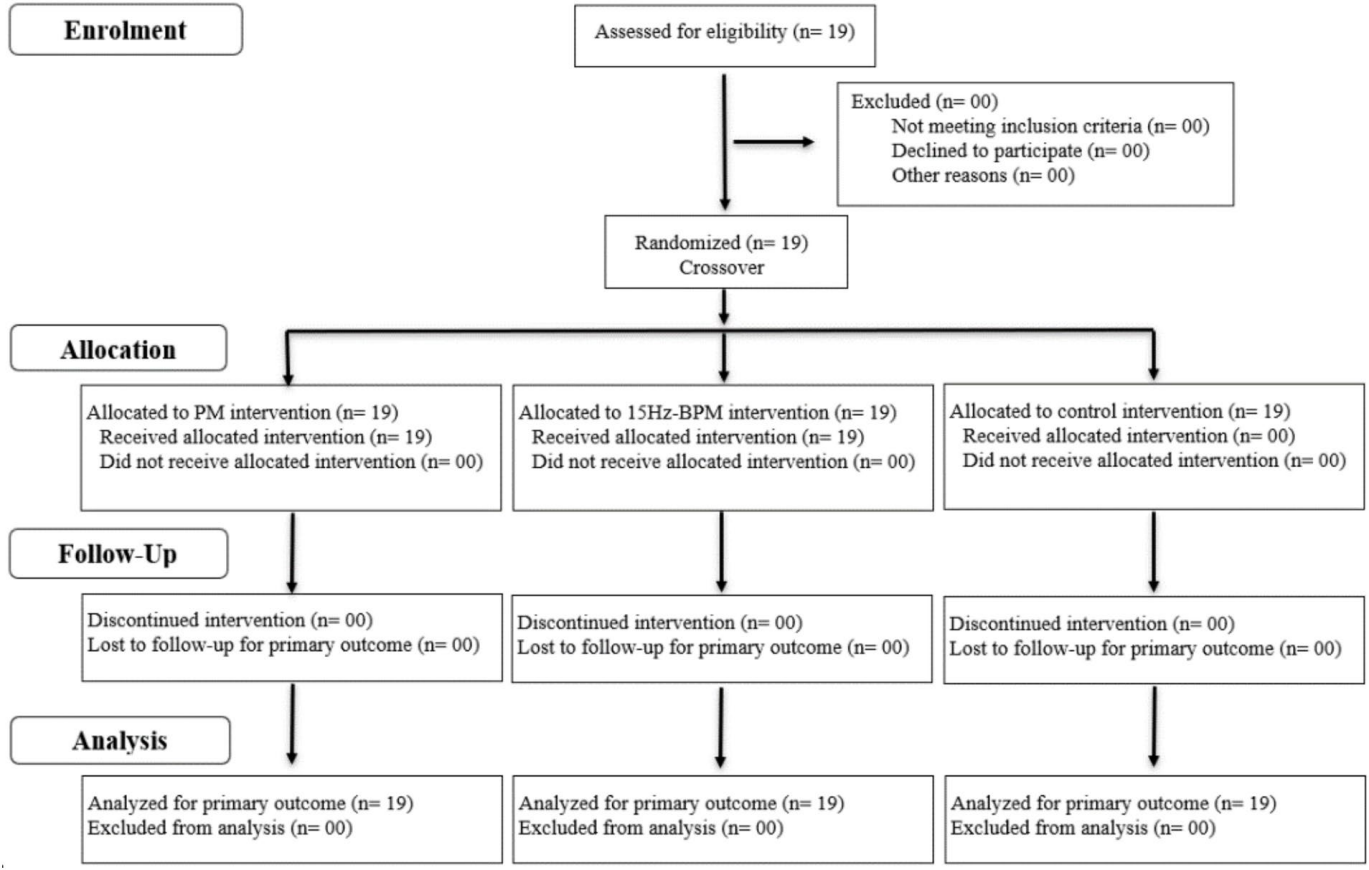
Consort flow diagram.

Nineteen kickboxers (Mean ± SD; age: 21.5 ± 1.8 years; body mass: 68.7 ± 5.3 kg; height: 1.80 ± 0.08 m; BMI: 21.2 ± 2.5 kg.m^−2^) volunteered to participate in the study. All kickboxers studied were amateur athletes competing kickboxing (full contact, light contact, low kick) within the same local training club. Inclusion criteria were regular training in three weekly physical training sessions (6 h/week). They had more than 4 years of kickboxing experience and at least 2 years of competitive experience. Exclusion factors were a recent history of muscular or joint injuries at least 6 months before experimental protocol, and absence at the time of the intervention. The Consolidated Standards of Reporting Trials (CONSORT) flow diagram is shown in Figure 2.

After a thorough explanation of the study’s objectives and potential risks, athletes provided written informed consent. The study adhered to the most recent Declaration of Helsinki guidelines for human research and received approval from the local ethics committee at the High Institute of Sport and Physical Education of Kef (0028/2024) prior to the commencement of data collection.

### 2.3 Musical characteristics

During familiarization sessions, kickboxers were asked to preselect their preferred instrumental music (without human singer) to be played during the test in the experimental protocol sessions. Each kickboxer was asked to select a piece of music that in their opinion would enhance motivation, reduce perceived exertion, and improve focus during recovery periods in order to maximize physical performance during simulated kickboxing combat. Using the Audacity software (http://www.audacityteam.org, accessed on December 2024), the tempo of each chosen song was adjusted to a fast tempo of 140 beats per minute (bpm) and set to a volume of 80 dB. Each song was played three times for a total of 3 min (1-min before round 1, 1-min before round 2, 1-min before round 3), through the same wireless headphones type (AirPods Pro 2, Apple, US) for all kickboxers. A tempo of 140 beats per minute (bpm) was selected for this study based on previous research demonstrating that fast music tempos are associated with better physical performance and induce more positive effects than slow tempo conditions in combat sports (Ouergui et al., 2023a; Jebabli et al., 2025).

With the preferred music, two pure tones with very slightly different frequencies were played separately in each ear. For example, a 440 Hz tone was played in the left ear and a 455 Hz tone in the right ear, which generated a 15 Hz binaural beat in the beta frequency range. Preferred binaural music at 15 Hz in the right and left ear was edited using Audacity software via Bluetooth. Binaural beat recordings were prepared by an independent researcher not involved in data collection or analysis to maintain analyst blinding. Given the inherent impossibility of kickboxer blinding to auditory interventions, standardized pre-session instructions emphasized focus on maximum effort regardless of audio condition to minimize expectancy bias. Data analysts remained blinded to condition codes throughout statistical analysis. During the no-music condition, headphones were worn, but no music was played.

### 2.4 Measurements

#### 2.4.1 Simulated combat on punching bag

**Figure 3:**
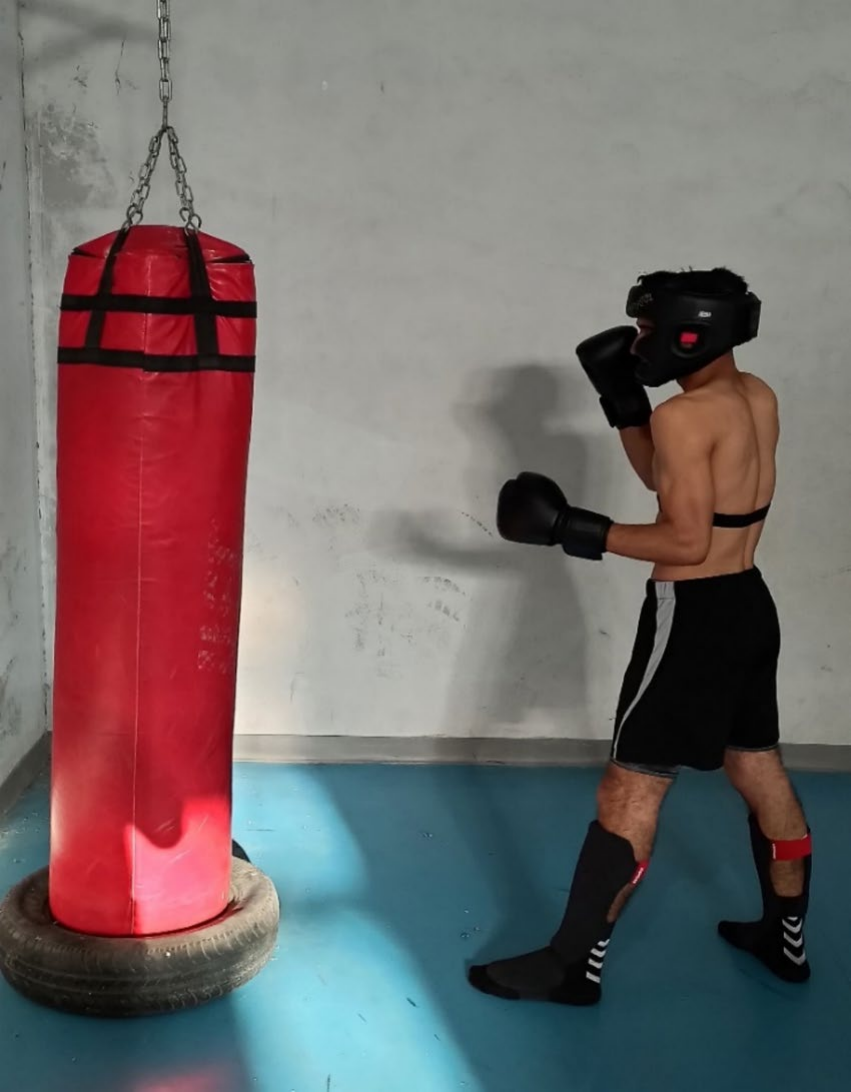
Simulated combat on punching bag.

The simulated combats on a punching bag consisted of three 2-min rounds with 1-min intervals passive recovery (see Figure 3). The number of rounds was conducted according to the official rules of the World Association of Kickboxing Organizations (Full contact, amateur competition). The simulated combats were conducted on a punching bag and not on a real simulation in order to ensure experimental control and minimize confounding variables such as opponent behavior, tactical choice, and interaction variability. The kickboxers were instructed to do their best performance in each round (all-out pacing strategy). Each kickboxer performed the combat on the same punching bag and with the same equipment (gloves, shin guards, mouth guards, etc.). All punch indices (type, number of impacts, peak velocity) were analyzed using Corner Boxing trackers (Corner wearables Ltd., UK), validated against high-speed video analysis (*r* = 0.92 for punch count, *r* = 0.88 for peak velocity). The devices demonstrated acceptable test-retest reliability (ICC = 0.89–0.94) across repeated testing sessions.

All kick indices (type, number of impacts of each technique, peak velocity) during the simulated combats were analyzed from video recordings using Kinovea software (version 0.9.5; France). Videos were recorded at 1080p resolution (1920 × 1,080, 16:9) and 48 frames per second (FPS) using the GoPro4 session camera. Two trained investigators, blinded to experimental conditions, independently analyzed kick techniques and velocities. Both investigators completed standardized training using 20 validation videos before data collection. Disagreements (<5% of observations) were resolved through consensus discussion with a third investigator. Inter-rater reliability demonstrated excellent agreement (ICC = 0.96 for kick count, ICC = 0.94 for technique classification, ICC = 0.93 for velocity measurements). During each round, a heart rate monitor (Polar Team 2, Polar Electro Oy, Finland) recorded both peak and average heart rates.

#### 2.4.2 Rating of perceived exertion

RPE was assessed using the Borg scale [6–20; (Borg, 1982)] after each round. This scale ranges from 6 (no exertion) to 20 (maximal exertion), with corresponding verbal anchors that progressively increase with perceived sensation intensity. After each round, kickboxers were asked three times about their RPE upper body, RPE lower body and RPE overall. The Cronbach’s alpha of the scale in the present study was 0.77.

#### 2.4.3 Feeling scale

Feeling scale assessed affective responses using an 11-point bipolar rating scale (−5 [very bad] to +5 [very good]) measuring current mood (Hardy and Rejeski, 1989). Kickboxers responded to “How are you feeling right now?” After each round. As a single-item measure, internal consistency analysis is not applicable; however, test–retest reliability in our pilot study showed acceptable stability (*r* = 0.74, *p* < 0.01) over 48-h intervals.

#### 2.4.4 Blood lactate

Three minutes after simulated combats, blood samples were taken from the fingertip (5 μL of blood) to measure blood lactate concentrations using a portable Lactate Monitor (lactate pro2, Akray, Japan).

### 2.5 Statistical analysis

Statistical assumptions were verified through Shapiro–Wilk normality tests and Mauchly’s sphericity assessments, with Greenhouse–Geisser corrections applied when sphericity was violated (*ε* < 0.75). Test–retest reliability for all variables was evaluated using Cronbach’s intraclass correlation coefficient (ICC) and the coefficient of variation (CV). To investigate condition and round effects, separate 3 (round) × 3 (condition) repeated-measures ANOVAs were conducted for each dependent variable cluster: (1) technical performance indices, (2) physiological markers, and (3) psychological responses. To control family-wise error rate across multiple comparisons, the Benjamini-Hochberg false discovery rate procedure was applied (*α* = 0.05). Post-hoc comparisons utilized Holm-Bonferroni correction for pairwise contrasts. Effect size was evaluated with partial squared where 0.01 < η_p_^2^ < 0.06 constitutes a small effect, 0.06 < η_p_^2^ < 0.14 a medium effect and η_p_^2^ > 0.14 a large effect (Cohen, 2013). Where the sphericity assumption was violated, the Greenhouse–Geisser adjustments of the *p*-values were reported. The level of significance was set at *p* < 0.05. All data analyses were performed using JASP v. 0.17.3 (University of Amsterdam, Amsterdam, Netherlands). Data were presented as means and standard deviations (SD).

## 3 Results

**Figure 4:**
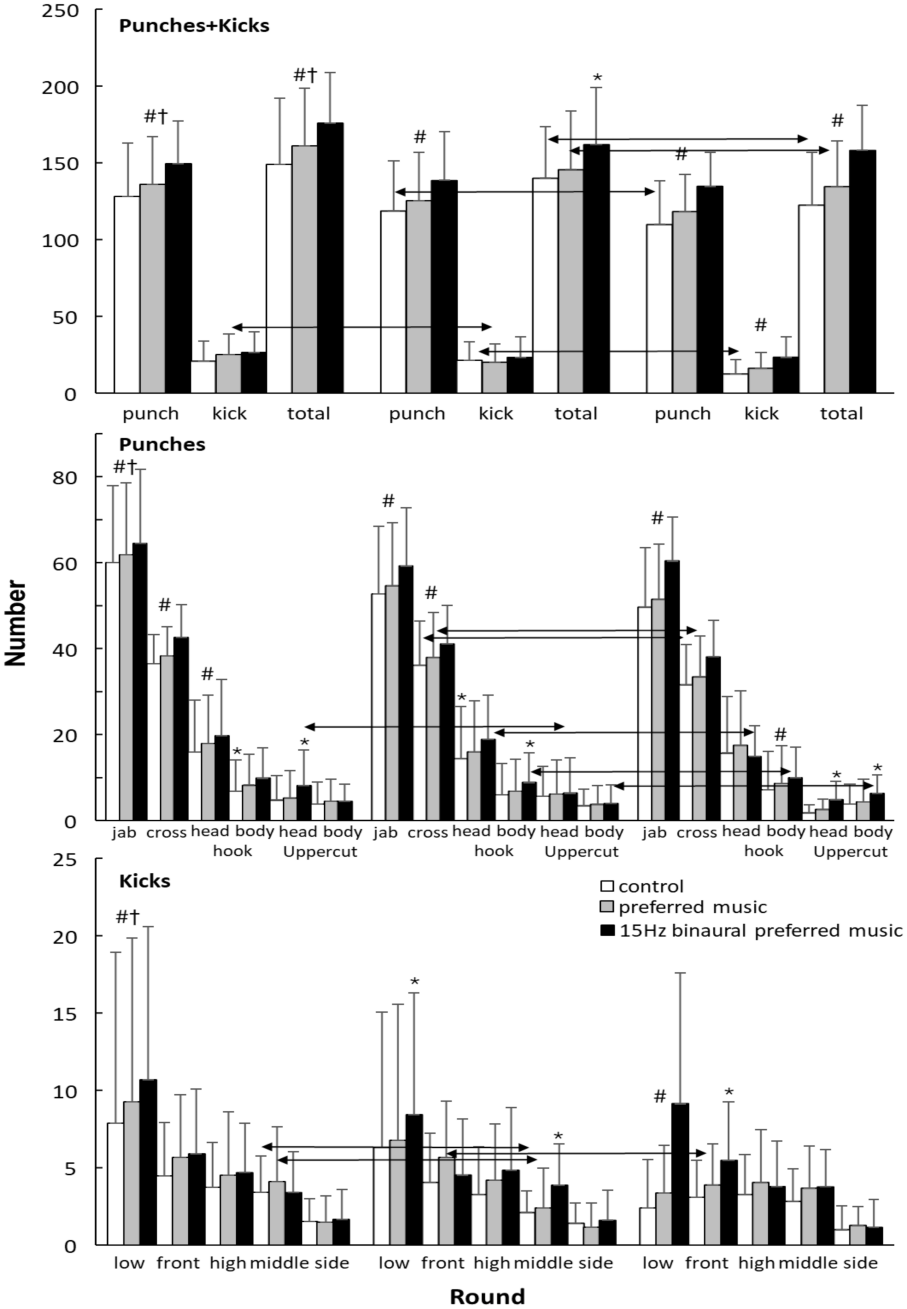
Average (±SD) number of punches and kicks per round under the three different conditions.

Significant effects of round, condition and round × condition interaction (*F* ≥ 7.4, *p* < 0.001, η_p_^2^ ≥ 0.29, 95% CI for mean differences: 2.1–4.8 impacts) were observed for total striking frequency. When specified in kicks and punches significant effects for round and condition (F ≥ 7.4, *p* < 0.001, η_p_^2^ ≥ 0.29) were found for both parameters, while for kicks also a significant interaction effect was observed (*F* = 5.5, *p* < 0.001, η_p_^2^ = 0.24). *Post hoc* comparison revealed that more impacts and specifically more punches were given in each round between all three conditions in which most were given in the 15 Hz-BPM condition, followed by the preferred music and control condition. For the kicks only these differences were clearly observed in round three (Figure 4). In addition, it was found that the number of impacts decreased significantly from round 1 to 2 and for the punches in each round (Figure 4).

When investigating the different types of punches and kicks during the different rounds and conditions it was found that rounds had a significant effect on all different types of punches, and only on the front and low kicks (*F* ≥ 3.6, *p* ≤ 0.038, η_p_^2^ ≥ 0.17). While a significant condition effect was found for all punches and for the front, middle and low kicks (*F* ≥ 3.3, *p* ≤ 0.048, η_p_^2^ ≥ 0.16) except for punches: hook head and uppercut body. Furthermore, a significant interaction effect was found for the same kicks as for the condition effect and all punches (*F* ≥ 3.7, *p* ≤ 0.008, η_p_^2^ ≥ 0.17), except the cross and hook body. Post hoc comparison revealed that most punches of all sorts were given during the 15 Hz-BPM, followed by the preferred music and control conditions, especially in the jabs, cross and hook head punches, which also mainly decreased from round 1 to two. For the kicks the low kicks showed the same development as in the punches, together with significant decreases in the front and middle kicks for control and preferred condition (Figure 4).

Physical performance parameters demonstrated significant condition, round, and interaction effects (*F* ≥ 9.1, *p* ≤ 0.001, η_p_^2^ ≥ 0.33). Post-hoc analysis revealed 0.3–0.5 mmol/L higher lactate concentrations in the control condition compared to music interventions (Cohen’s d = 0.6–0.8), indicating practically meaningful differences in metabolic demand. Peak punch and kick velocity had a different development over the three rounds between the three conditions. So did the control condition decreased impact velocity in round three compared to the other two rounds, while in the preferred music and 15 Hz-BPM conditions it first increased from the first to the second round and decreased again from the second to the third round, while this decrease was significantly less for the 15 Hz-BPM condition compared with the preferred music condition. Average and peak heart rate increased for all conditions from round to round. However, in the 15 Hz-BPM the mean and peak heart rates increased more than in the other conditions (see Table 1).

**Table 1:** Peak ± SD kicking and punch velocity, average and peak heart rate during each round and lactate concentration after three rounds for each condition.

	Peak velocity (m/s)		Heart rate (pulse/min)		Lactate
	Punch	Kick	Average^#^	Peak^#^	(mmol/L)
Round 1					
Control	22.2 ± 2.1	27.3 ± 2.4	166.5 ± 4.6	181.9 ± 4.0	NA
Preferred music	22.1 ± 2.0	27.3 ± 2.4^†^	165.8 ± 4.6	181.3 ± 3.3	NA
15 Hz condition	22.2 ± 2.1^†^	27.4 ± 2.4^*†^	167.0 ± 5.3	182.6 ± 3.3	NA
Round 2					
Control	22.2 ± 2.1^*^	27.3 ± 2.4^*^	168.5 ± 4.6*	183.9 ± 4.0	NA
Preferred music	22.3 ± 2.1^*†^	27.4 ± 2.4^*†^	170.4 ± 4.3	185.1 ± 3.7	NA
15 Hz condition	23.6 ± 2.1^*†^	28.6 ± 2.4^*†^	171.4 ± 4.7	187.1 ± 2.9^*^	NA
Round 3					
Control	21.8 ± 2.1^*†^	27.0 ± 2.4^*†^	170.9 ± 4.1	186.3 ± 3.5	6.1 ± 0.8^*^
Preferred music	22.1 ± 2.0^*^	27.2 ± 2.4^*†^	170.8 ± 4.5	186.6 ± 4.1	5.8 ± 0.7
15 Hz condition	22.7 ± 2.0^*†^	27.7 ± 2.4^*†^	175.4 ± 4.7^*^	190.2 ± 2.4^*^	5.8 ± 0.6

^*^ indicates a significant difference with the other two conditions for this round.

^†^ indicates a significant difference with the other two rounds for this condition.

^#^ indicates a significant increase for all conditions from round to round.

The psychological parameters were also all significantly affected by rounds and condition (*F* ≥ 4.0, *p* ≤ 0.027, η_p_^2^ ≥ 0.18), while only a significant interaction effect was found for feeling and upper body RPE (*F* ≥ 4.1, *p* ≤ 0.005, η_p_^2^ ≥ 0.19). Post-hoc analysis revealed progressive RPE increases across rounds, with the 15 Hz-BPM condition demonstrating 1.1–1.4-point reductions compared to control in round 3. These differences exceed the minimal clinically important difference of 1.0 point established for RPE scales in exercise research, indicating practically meaningful reductions in perceived exertion. For the feeling grade in increase from round 1 to 2 was found (but not significant per condition), while in round three the feeling grade was significantly higher in the 15 Hz preferred condition compared to the other two conditions (see Table 2).

**Table 2:** Mean ±SD rate of perceived exertion and feeling after each round for each condition.

	Rate of perceived Exertion (6–20)			Feeling scale
	Upper body^#^	Lower body^#^	Overall^#^	(−5 to +5)
Round 1				
Control	11.2 ± 1.5	11.6 ± 2.1^*^	12.0 ± 2.1	1.1 ± 0.6
Preferred music	10.8 ± 1.4	10.2 ± 1.9	11.3 ± 1.6	1.2 ± 0.5
15 Hz condition	10.2 ± 2.0	9.5 ± 1.5	10.1 ± 1.2^*^	0.9 ± 0.8
Round 2				
Control	15.7 ± 1.3^*^	15.1 ± 1.6*	15.6 ± 1.3^*^	1.2 ± 0.6
Preferred music	14.2 ± 1.6	13.4 ± 1.9	14.6 ± 1.9	1.6 ± 0.8
15 Hz condition	14.1 ± 2.3	12.6 ± 2.3	14.2 ± 2.2	2.1 ± 1.6
Round 3				
Control	18.5 ± 1.1	18.3 ± 1.6	18.9 ± 1.1	0.9 ± 0.7
Preferred music	18.4 ± 1.5	17.8 ± 1.5	18.7 ± 1.3	1.2 ± 1.4
15 Hz condition	17.4 ± 1.5^*^	16.2 ± 12.6^*^	17.5 ± 1.6^*^	2.4 ± 1.7^*^

^*^ indicates a significant difference with the other two conditions for this round.

^#^ indicates a significant increase for all conditions from round to round.

## 4 Discussion

The present study examined the effect of listening to preferred music and 15 Hz Binaural beat, on activity profile and psychophysiological responses during a simulated kickboxing combat. As hypothesized, the results show that 15 Hz-BPM, during pre- and inter-round recovery, exerts significant positive effects on combat cadence and velocity, following PM condition, compared to the control condition. Furthermore, 15 Hz-BPM could be more effective strategy to improve heart rate and feeling scale compared to other conditions. In addition, both 15 Hz-BPM and PM conditions produced lower RPE and lactate values post-round than the control condition.

The present study showed that the number of punches and kicks thrown was significantly higher at 15 Hz-BPM than in the other conditions. Similarly, kickboxers exhibited higher peak velocities at 15 Hz-BPM than PM and control conditions (Agrebi et al., 2024). Velocity initially increased from the first to the second round for all three conditions and then decreased again in the third round. In the control and PM conditions, velocity returned to or was lower than in the first round, while remaining higher than 15 Hz-BPM, which also resulted in higher peak velocities than in the other two conditions (see Table 1). Collectively, this indicates cumulative performance gain from the synergistic application of preferred music and beta binaural beats during brief recovery periods, in favor of the efficacy of such interventions in combat sports competition.

Interestingly, the contrasting findings with Wang et al. (2024), who observed no performance benefits from beta binaural beats in soccer players, likely reflect fundamental differences in exercise modality and intervention timing. Wang et al. (2024) applied binaural beats solely during warm-up for continuous sprint performance, whereas our study implemented inter-round recovery applications during intermittent high-intensity exercise. Thus, combat sports’ distinct neuromuscular demands, requiring precision timing, coordination, and decision-making under fatigue, may be more responsive to beta frequency entrainment effects compared to the primarily metabolic demands of repeated sprinting.

Importantly, the present study brings new information by showing that this combined effect benefits recovery during maximal efforts in male kickboxers, not only during warm-up phases as described before. However, these results could not be extrapolated to female kickboxers, who would behave differently when faced with music stimulation depending on the variation in hormonal profiles, autonomic regulation and affect processing according to sex (Jebabli et al., 2025; Goshvarpour et al., 2013).

From a neurophysiological perspective, the observed performance improvements may result from established neurophysiological mechanisms documented in music exercise research (Dhahbi et al., 2024; Souissi et al., 2025). Music interventions consistently activate the left inferior frontal gyrus and insular cortex while attenuating fatigue-related neural processing (Ballmann, 2021). The additional benefits of 15 Hz binaural beats likely reflect documented cortical entrainment effects, where beta frequency stimulation enhances attentional networks and sensorimotor integration (Beauchene et al., 2016; Souissi et al., 2025).

Nevertheless, these interpretations are speculative, as no direct neurophysiological data were collected in this research. In the same context, previous research on working memory and cognitive load has indicated that 15 Hz binaural beats are linked to states of active arousal, concentration, and heightened vigilance (Basu and Banerjee, 2023), activating neural networks of fine motor control and central fatigue resistance during and after cognitive exercise (e.g., the Time Load Dual Back test) (Engelbregt et al., 2021; Wang et al., 2022).

In addition, it is also interesting to note that the increase in physical performance observed in each round, during 15 Hz-BPM condition, is accompanied with lower perceived fatigue in kickboxers, as evidenced by the lower values of the blood lactate and RPE after the combat simulation. The physiological marker, particularly blood lactate, presented in this study provides an objective method for explaining physical fatigue. In fact, lower blood lactate concentration post combat, during music conditions, is a sign of better metabolic efficiency and faster recovery (Jebabli et al., 2020; Ballmann, 2021). Consistently, both studies have shown that music enjoyed during exercise has a relaxing effect, leading to decreased muscle tension, improved blood flow and lactate removal, and reduced lactate production in active muscles (Jebabli et al., 2023).

On the other hand, listening to 15 Hz-BPM before and during inter-round recovery increases average and peak heart rates in each round, associated with an increase in power output, is well supported by previous studies (Ouergui et al., 2023b; Delleli et al., 2024). However, these results are partly contradicted by other studies that found no significant difference in heart rate with listening to preferred fast-tempo music before and during aerobic exercise (Nakamura et al., 2010; Dyer and McKune, 2013). These inconsistencies may be explained by methodological differences in experimental protocols, study population type, gender differences and musical characteristics (Ballmann, 2021).

Regarding psychological responses, RPE, in both lower and upper body, were significantly lower in PM and the 15 Hz-BPM conditions compared to control condition during round 1 and round 2. In the third round, RPE levels were significantly lower only in 15 Hz-BPM condition compared to the other conditions. These findings are consistent with the idea that the combination of music binaural beats can reduce perceived exertion, perhaps by attentional distraction mechanisms.

Moreover, during the third round, the feeling scale was perceived positively, with scores during the 15 Hz-BPM condition being significantly higher compared to the other conditions (see Table 2). This positive mood sustenance might be essential to sustaining effort in repeated high-intensity efforts. These results could represent a key mechanism for improving physical performance, as reported in previous research (Ouergui et al., 2023b; Jebabli et al., 2025).

In contrast to studies that specifically analyzed warm-up music only, our study shows that motivational benefit is particularly found during recovery where music is paired with binaural beats. Future research will be required to explore the effect of frequency of beats, tempo, and individual liking on mood regulation.

## 5 Limitations and future study

Some limitations should be noted. First, the lack of neurophysiological measurements, such as the absence of electroencephalography or heart rate variability data, limits mechanistic interpretations of the observed effects. Logistical problems encountered during field research in combat sports justify these exclusions. Second, despite the use of a punching bag rather than a human partner to neutralize certain external influences (e.g., tactical variations), this diminishes the ecological validity of the study. Third, the sole participation of male kickboxers makes the results not generalizable to female kickboxers.

Future studies might consider investigating the effects of preferred music and different binaural beat frequencies, namely theta and gamma, on physical performance, while further exploring neurophysiological and cognitive responses (e.g., electroencephalography, heart rate variability, working memory, attention tasks) in male and female kickboxers. These physiological and cognitive measures should be a part of an integrated approach to better understand the neurocognitive mechanisms underlying the combined effects of binaural beats and music. Furthermore, it is uncertain whether the positive effects observed on mood, RPE, and physiological parameters with a 15 Hz heart rate are transient or constant across recovery trajectories. These effects need to be verified in future studies.

## 6 Conclusion

This study provides preliminary evidence supporting the use of a combination of 15 Hz binaural beats and preferred music as a recovery intervention in simulated combat conditions. These data are based on significant improvements in punch frequency and velocity, as well as positive psychophysiological responses. Therefore, coaches and practitioners can attempt to implement this type of auditory stimulation into training and competition recovery to enhance psychophysiological readiness and uphold high standards of performance across repeated rounds. This is one such easy, non-invasive, and easy to implement in practice solution that has both performance and psychological benefits for athletes.

## Data Availability

The original contributions presented in the study are included in the article/Supplementary material, further inquiries can be directed to the corresponding author.
